# Motor Representation of Actions in Children with Autism

**DOI:** 10.1371/journal.pone.0044779

**Published:** 2012-09-10

**Authors:** Giuseppe Cossu, Sonia Boria, Cristiana Copioli, Roberta Bracceschi, Virginia Giuberti, Erica Santelli, Vittorio Gallese

**Affiliations:** 1 Dipartimento di Neuroscienze, Università di Parma, Parma, Italy; 2 Unità di Neuropsichiatria Infantile, AUSL Reggio Emilia, Reggio Emilia, Italy; 3 IIT (Italian Institute of Technology) Brain Center for Social and Motor Cognition, Parma, Italy; Centre Hospitalier Le Vinatier (Bât. 452), France

## Abstract

**Background:**

Children with Autistic Spectrum Disorders (ASD) are frequently hampered by motor impairment, with difficulties ranging from imitation of actions to recognition of motor intentions. Such a widespread inefficiency of the motor system is likely to interfere on the ontogeny of both motor planning and understanding of the goals of actions, thus delivering its ultimate effects on the emergence of social cognition.

**Methodology/Principal Findings:**

We investigate the organization of action representation in 15 high functioning ASD (mean age: 8.11) and in two control samples of typically developing (TD) children: the first one, from a primary school, was matched for chronological age (CA), the second one, from a kindergarten, comprised children of much younger age (CY). We used nine newly designed behavioural motor tasks, aiming at exploring three domains of motor cognition: 1) imitation of actions, 2) production of pantomimes, and 3) comprehension of pantomimes. The findings reveal that ASD children fare significantly worse than the two control samples in each of the inspected components of the motor representation of actions, be it the imitation of gestures, the self-planning of pantomimes, or the (verbal) comprehension of observed pantomimes. In the latter task, owing to its cognitive complexity, ASD children come close to the younger TD children’s level of performance; yet they fare significantly worse with respect to their age-mate controls. Overall, ASD children reveal a profound damage to the mechanisms that control both production and pre-cognitive “comprehension” of the motor representation of actions.

**Conclusions/Significance:**

Our findings suggest that many of the social cognitive impairments manifested by ASD individuals are likely rooted in their incapacity to assemble and directly grasp the intrinsic goal-related organization of motor behaviour. Such impairment of motor cognition might be partly due to an early damage of the Mirror Neuron Mechanism (MNM).

## Introduction

Children’s aloneness from the beginning of life is a marking trait of autistic disturbances. Indeed, as Kanner pointed out, “the children’s inability to relate themselves in the ordinary way to people and situations from the beginning of life” [1] represents “the outstanding, pathognomonic, fundamental disorder” in Autistic children. However, this feature of infantile autism is embedded within a cluster of concomitant disorders, frequently involving the motor domain. In particular, as Leo Kanner, again, reported: “almost all mothers [.] recalled their astonishment at the children’s *failure to assume at any time an anticipatory posture* preparatory to being picked up”. Yet, the relevance of motor disorders in autistic children was downplayed for years, since the varieties of motor impairments are camouflaged by an overwhelming lack of social and emotional reciprocity. Furthermore, no theoretical frame was available in the early studies on motor impairment that could reliably intersect the failure of anticipatory postures, stereotyped behaviours, imitation deficits, clumsy gait, and other motor impairments with the impairments of sociability and communication [2].

These difficulties notwithstanding, it became increasingly clear that motor disorders were a core component of the Autistic disorder. More than 30 years ago, Marian De Meyer suggested that “body imitation failure” [3] might be a crucial factor in precluding interpersonal communication in Autism. Subsequent research further corroborated these early findings, as documented in the seminal review by Rogers and Pennington [4]. These Authors reckoned strong evidence for an imitation deficit in Autism, thus leading them to postulate a developmental model of Autism in which “early cascading social-communicative impairments might stem from an early deficit in motor imitation”. A vast body of subsequent research further increased the robustness of the motor hypothesis [2]–[5]. Yet, some studies yielded contradictory findings, thus leading to question the notion of “imitative deficit” in children with autism. A study by Whiten and Brown [6], for instance, analysed different categories of imitative tasks in a sample of ASD patients, ranging from young age to adolescence and adulthood: their results did not find evidence to support a general deficit of imitation in autism. Indeed, a survey of the current literature on this issue [7], brings to light more than one dissonant voice, concerning the disputed occurrence of imitation disorders in ASD children [8], [9] as well as the interpretation of the observed impairments in imitation [10]. In particular, it has been shown that, in adult subjects with ASD, the imitation scores are not different from controls, when participants are instructed to attend to imitation-relevant parts of the stimuli [8]. Furthermore, it appears that the ability to imitate other’s actions is unlikely to be grounded on a simple, unitary skill, being it significantly influenced by external variables such as the use of familiar vs. unfamiliar objects [11], or by the relevance of sensory feedback [12]. Accordingly, it has been aptly focussed the need for a distinction between mimicry and imitation as well as for the adoption of a “comparative taxonomy of imitation and a standardized methodology across researchers” [7]. Thus, it is no surprise that the gulf between the description of imitation deficits in Autism and an explanation of the mechanisms underlying social-communicative breakdown in Autistic children is still very large.

Until recently, explanatory hypotheses of the motor impairment in Autistic children were, at best, tentative, as the core neurophysiologic mechanisms of the syndrome remained obscure. However, recent advances in the neurophysiology of intentional actions have begun to shed some light on the intersection between the action system and Autism. Such a new step was provided by the discovery, in the ventral pre-motor cortex (area F5) of the macaque monkey, of a class of motor neurons, mirror neurons that discharge both during the execution and the observation of goal-directed motor acts [13], [14]. Subsequently, mirror neurons were also localized in the inferior parietal lobule, reciprocally connected with area F5 (PF/PFG mirror neurons) [15], [16]. A further step forward in the research on the mirror neuron mechanism (MNM) emerged from the discovery that the parietal mirror neurons, besides coding the goal of a single executed/observed motor act, like grasping an object, code the overall *action intention* as well (e.g., bringing the grasped object to the mouth or into a container [17]. The MNM maps integrated sequences of goal-related motor acts (grasping, holding, bringing, placing), defined as the different “words” of a “motor vocabulary” [18], [19], clustering them into “syntactically” separate and parallel intentional “action sentences”. These results suggest – at least at such a basic level – that the “core intention” of eating or placing the food is also coded by parietal mirror neurons.

The relevance of the mirror mechanisms was further enhanced by their phylogenetic intrusion into the species of homo sapiens. It has been documented from both indirect measures [16], [20], [21] as well as from direct extracellular recordings of neural activity in neurosurgical patients [22] that a sensory-motor mirroring mechanism involving homologue cortical areas is also present in the human brain. The MNM in humans is directly involved in imitation of simple movements [23], imitation learning of complex skills [24], in the perception of communicative actions [25], and in the detection of basic action intentions [26]. This latter study showed that premotor mirror areas, previously thought to be involved only in action recognition are actually also involved in understanding the “why” of action, that is, the motor intention promoting it.

The results of Iacoboni et al. [26] suggest that most of the time – at least at the level of basic intentional actions – even humans do not explicitly represent intentions as such, when understanding them in others. By means of embodied simulation [27], [28], when witnessing others’ behaviours the motor intentional contents of the observed agent can be directly grasped by the observer without the need of representing them in propositional format. Thus, converging evidence from these studies suggests that the MNM underlies the multifaceted sides of action representation and substantial aspects of social interaction. Accordingly, a growing attention has recently been directed toward the intersection between the MNM and the disruption of social interaction in Autism [29]. The relevance of the MNM for the Autistic syndrome stems precisely from the fact that we have now a grasp on a neural mechanism allowing a parsimonious solution to the problem of matching the observed (and heard) action, which is still meaningless at the sensory stage, into a meaningful motor event for the observer. In other words, mirror neurons likely provide a pre-cognitive, direct *understanding* of the observed action [13], [16]. We reasoned that this newly acquired knowledge on the motor system, that we dubbed *motor cognition hypothesis* [30], might indeed provide a powerful tool for deciphering the role of motor disorders in Autism.

It is unlikely, though, that autism can be exclusively reduced to a deficit of motor cognition. Even less likely autism can be simply equated to a mere absence of the MNM. Both views appear too simplistic and fall short of capturing the multi-layered and diversified aspects characterizing ASD. Indeed, there is clear evidence that failure of ASD individuals in imitation of transitive actions extends beyond mere motor-executive aspects, by involving non-imitative aspects as well [10]. Yet, all of these “non-imitative” aspects are concerned with the motor domain, although at the more abstract planning levels of intentional actions. We posit that many of the social cognitive impairments manifested by ASD individuals are rooted in their incapacity to assemble and directly grasp the intrinsic goal-related organization of motor behaviour. By viewing this issue from a developmental perspective, we reasoned that the daily experience with common objects might provide ASD children, albeit slowly, with a rudimentary archive of stored actions that might be prompted (and facilitated) by the mechanism of *affordance* when the child attempts to grasp the object for a spontaneous action or for imitation. Hence, in our experimental design, we introduced some tasks requiring the imitation of conventional actions with objects and contrasted them with other tasks that required either imitation of conventional actions *without* objects or of unconventional actions with objects. Accordingly, we set up an experimental design tailored at exploring, in the same child, some of the components that constitute the basic architecture of motor cognition: action imitation, self-generation and comprehension of pantomimes. We expect that an integrated analysis of these three domains of motor cognition may eventually provide a better understanding of the core mechanisms of autism.

## Methods

### Participants

All of the children participating to this study completed the set of the presented tasks that had been previously approved by the local ethical committee (Comitato Etico Unico per la Provincia di Parma) and were conducted according to the Helsinki declaration. Written informed consent was obtained from the parents of each child involved in our study.

We examined a group of fifteen high functioning children (13 males and 2 females; mean age: 8.11 (SD 1.9) with autism or autism spectrum disorder recruited from a Regional Centre for Autism in Northern Italy. All patients were free from any evident neurological abnormalities as well as from hearing or visual impairment. On the Wechsler Intelligence Scale for Children-Revised (WISC-R) [31] they obtain a mean performance IQ of 95.8 (SD = 14.84), a mean verbal IQ of 86,8 (SD = 14,69) and a mean Total IQ score of 91 (SD 12.02).

The diagnosis was made according to the clinical criteria of DSM-IV [32] and to the Autism Diagnostic Observation Schedule (ADOS) [33]. The module 2 and the module 3 of the ADOS were used to confirm a diagnosis of autism or autistic spectrum disorder. Based on the results of these scales and clinical judgment, 9 out of 15 children met the criteria for autistic disorder, and the remaining 6 met the criteria for autism spectrum disorder. Henceforth, we will refer to the whole clinical sample as children with Autistic Spectrum Disorder (ASD).

We selected two control samples of TD children: the first one matched for chronological age (CA) and the second one of a chronologically younger age (CY). The first sample comprised 21 children from primary schools with a mean age of 8.7 years (SD 0.3); the second sample included 42 TD children from kindergarten (mean age of 4,9 SD 0.3). TD controls had no history of neurological or psychiatric diagnosis and were recruited from local primary and maternal school, according to a balanced match between males and females. Their non-verbal cognitive level, assessed by means of the Raven’s Progressive Matrices [34], was within the normal range. The CA and the CY samples had a mean IQ score of 90,0 (SD 8,5) and 89,5 (SD 11,5), respectively; a t-test showed no significant difference either between the ASD children and the CA sample (t = 0.3003; p<0.765), or between the ASD children and the CY sample (t = 0.424; p<0.672).

### Measures and Procedures

We set up three sets of tasks for the assessment of imitation, self-generation and comprehension of actions, respectively; the whole project comprised 9 different tasks. Each child was required to inspect a digitized video-clip and to perform (or recognize) an action according to the received instructions.

#### Imitation of actions

The battery consisted of six tasks, each comprising 15 items.

1. *imitation of conventional actions with an object*: the child watches an actor making an action with an object (e.g. drinking from a glass), then the child is required to repeat the observed action with a real object placed on the table, immediately after viewing the video clip.
2. *imitation of conventional actions without objects*: the actor mimes an action without any object (e.g.: acting as if drinking from a glass without the glass), then the child is asked to repeat the action without the object.
3. *imitation of non conventional actions with an object*: the child watches an actor making a non conventional action with an object (for example: turn a book around with the elbow), the child is asked to do the same.
4. *imitation of finger position*: the items are performed with one or both hands; the child observes on the screen a particular hand posture being produced by the examiner with either one or both hands (e.g. extending or flexing one or more fingers); then the child is required to repeat the observed finger position.
5. *imitation of oro-facial gestures* devoid of emotional content: the child watches an actor making a facial grimace, or protruding the tongue, then the child is required to repeat the observed gesture.

#### Production of pantomimes

The battery consisted of two tasks, each comprising 15 items.

1. *production of a pantomime from the picture of a tool*: the child watches the drawing of an object/tool on the screen and then he/she is required to show how the real object/tool is commonly used (e.g. by viewing a hammer, the child has to mime hammering).
2. *production of a pantomime from the oral naming of a tool*: the actor says the name of an object/tool and the child is required to show how the object/tool is commonly used (e.g.: the actor says “glass” and the child has to mime drinking from a glass).

#### Comprehension of pantomimes

The battery comprised two tasks, each one including 15 items.

1. *Visual Comprehension of Pantomimes*: an actor mimes an action without the object; soon afterwards, the child views on the screen three pictures representing three tools (one target and two foils) and is required to point to the target picture, related to the mimed action.
2. *Oral Comprehension of Pantomimes*: an actor says the name of an object/tool and mimes an action either representing the correct use of the object/tool or an action which is unrelated to the object/tool; the child is required to judge whether the mimed action corresponds correctly to the named object.

The order of presentation of the tasks was counterbalanced across participants and the responses were video-recorded for further inspection. No time constraints were imposed, although we recorded the total time for each task.

We rated the children’s actions according to the scoring criteria outlined by Watkins, Dronkers & Vargha-Khadem [35]. In particular, each movement was rated on a scale from zero to three points: zero for no movements at all or for a plainly incorrect imitation; one point for an attempt at the correct imitation but poor execution; two points for a correct movement with minor problems in execution and three points for a correct execution of the imitation required. In the two tasks assessing the Comprehension of Pantomimes, each response was scored either zero (for an incorrect pointing/judgement), or one, for a correct response.

In order to obtain a reliability measure of the scoring procedure for the imitation and production tasks, the children’s performances were rated by two independent clinical psychologists. Inter-rater reliability was then calculated: the findings show a Pearson correlation score of *r* = .0825 (p<.000) for the imitation of conventional actions with objects; for all of the remaining 6 motor tasks the *r* score ranges from.0928 (p<.000) to.9887 (p<.000).

## Results

We will present our findings from the nine batteries by clustering the results around the domains of imitation, production and comprehension of pantomimes. For each domain we will systematically compare ASD children with the two control samples of TD children: a chronological age-matched (CA) and a younger sample (CY), respectively. In particular, we will first compare imitation of conventional actions with objects vs. conventional actions without objects; our aim is to explore, in ASD children, whether the context might play a facilitating role in imitation, analogous to the positive effect of context on intention understanding [36], that we reported, in a previous study A comparison of non-conventional vs. conventional actions with objects aims to explore the planning of “new” actions, unconnected with the routine use of the objects, thus minimizing the facilitating role of the context. Likewise, we will compare the imitation of facial gestures and the imitation of finger position, since both tasks are devoid of proper meaning, as they are not goal-directed.

Even a cursory glance at the findings makes it clear that neither a normal level of intelligence nor an advantage of four years discrepancy (with the younger sample), suffices for the Autistic children to outperform even their younger siblings in any of the motor tasks they were presented with.

### Imitation

**Figure 1:**
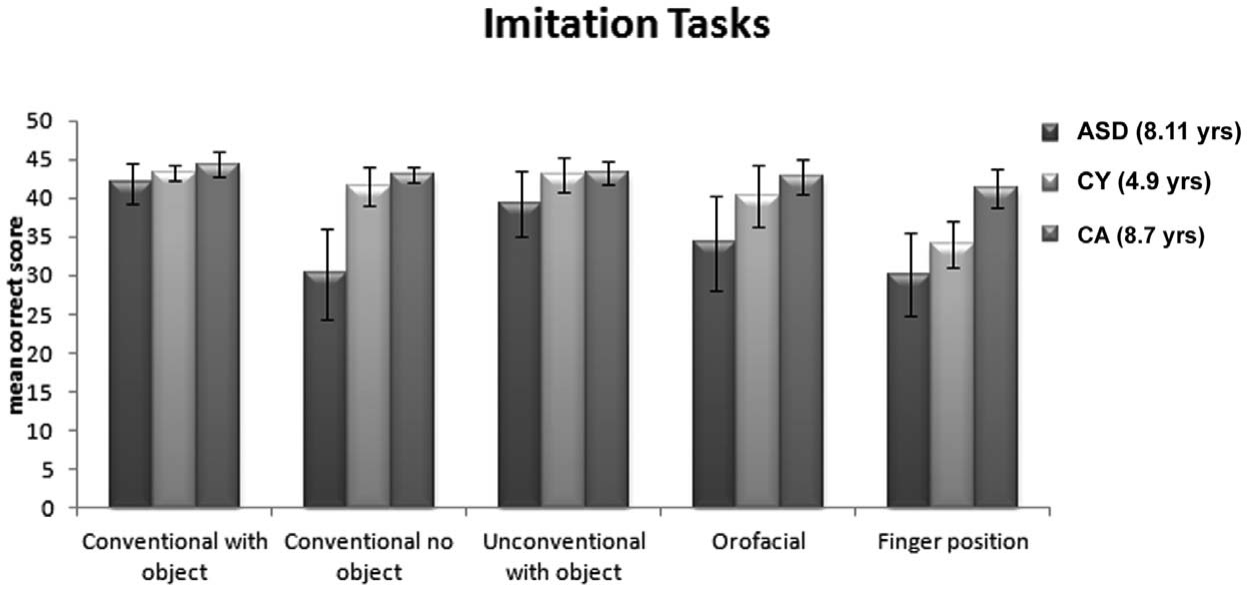
Imitation Tasks. Mean correct responses and standard deviations in the five sets of imitation tasks.

ASD children perform significantly worse than the TD samples in the imitation tasks of conventional actions. We assessed the role of the presence/absence of objects (within-subject variables) in the three groups (between-subject variables) by means of a mixed-model design ANOVA. The results indicate that Group (ASD and the two TD groups) and Task (with and without objects) as main factors, are highly significant: (Group: F [2,75] 72,55 p<.0001; Task F [1,75] 220,52 p<.0001). Likewise significant is the interaction of Group by Task (F [2,75] 68,27, p<.0001). In order to provide a systematic comparison of the degree of efficiency of the three samples for each one of the two types of tasks, we also run a Univariate ANOVA: the results show that the differences in the Imitation with objects is highly significant (F [2,75] 16,02, p<.0001) across the three groups; similarly significant are the differences in the imitation task without objects (F [2,75] 82.27, p<.0001). Interestingly, a post hoc test (Bonferroni), indicates that the ASD children achieve significantly lower levels of performance when compared with both the younger and the age-matched controls in the imitation with object (p<.0001) as well as in the imitation without object (p<.0001). Furthermore, for the ASD children, the relevance of the objects in the imitation tasks is plainly attested by the degree of the within-sample discrepancy between the imitation task with objects (mean correct: 42 -SD 2.61-) and without objects (mean correct: 30.26 -SD 5.89-). A quite different picture emerges from the within sample comparison between the two tasks in the CY children (mean correct 43.35 -SD 1.02- *vs*. 41.47 -SD 2.50) and in the CA sample (mean correct 44.35 -SD 1.61- *vs.* 43.05 -SD 1.04) (Fig. 1) for the object/no-object tasks, respectively.

When the ASD children are required to imitate non-conventional actions with objects, the need for planning a new (unconventional) action again penalizes their performances. A mixed-model design ANOVA, with Group (ASD and the two TD control groups) and Task (conventional *vs.* non-conventional actions with objects) as main factors, indicates that both factors are highly significant: (Group: F [2,75] 27,61, p<.0001; Task F [1,75] 22,90, p<.0001); likewise significant is the interaction of Group by Task (F [2,75] 3,53, p<.034). A Univariate ANOVA show that the differences in the imitation of conventional actions is significant ((F [2,75] 16,02, p<.0001) across the three groups; similarly significant are the differences in the imitation of non conventional actions (F [2,75] 18,63, p<.0001). A post-hoc test (Bonferroni) indicates that the ASD children fare at a significantly lower level with respect to both the CA controls (p<.0001) and the CY controls (p<.0001).

The results from the last two imitation tasks (finger position and oro-facial gestures) further corroborate the previous findings (Fig. 1). A mixed-model design ANOVA, with Group (ASD and the two TD samples) and Task (finger position and oro-facial gestures) as main factors, indicates that both factors are highly significant: (Group: F [2,75] 50,88, p<.0001; Task F [1,75] 36,72, p<.0001), as well as the interaction of Group by Task (F [2,75] 5,38 p<.007). In order to provide a systematic comparison of the degree of efficiency of the three samples for each one of the two types of tasks, we also run a Univariate ANOVA: the results show that the differences in the Imitation of finger position is highly significant (F [2,75] 45,62, p<.0001) across the three groups; similarly significant are the differences in the Imitation of oro-facial gestures (F [2,75] 20,51, p<.0001). A post-hoc test (Bonferroni), further corroborates these findings by showing that the ASD children yield significantly lower levels of performance when compared with both the younger and the age-matched controls in the imitation of finger positions (p<.0001) as well as in the imitation of oro-facial gesture (p<.0001).

### Production of Pantomimes

**Figure 2:**
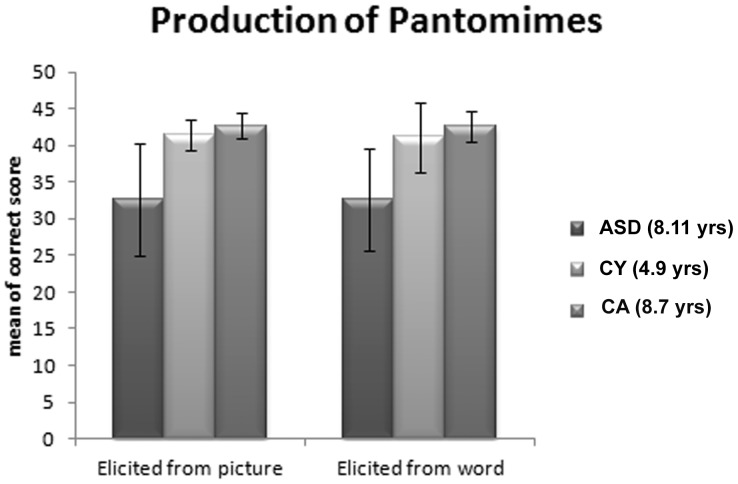
Production of Pantomimes. Mean correct responses and standard deviations in the two tasks elicited by either a picture of the tool, or by the spoken word thereof.

In the two tasks requiring the production of pantomimes (prompted by a visual image or by a spoken name, respectively) the discrepancy between the ASD children and the TD control samples remains remarkable (Fig. 2). A mixed-model design ANOVA indicates that the Group Factor (ASD and the two TD samples) is highly significant: (F [2,75] 38,61, p<.0001), whereas neither the Task Factor (pantomimes elicited from visual and verbal cues) (F [1,75] 0,205, p<.652) nor the interaction of Group by Task (F [2,75] 0,051, p<.950) is significant.

### Comprehension of Pantomimes

**Figure 3:**
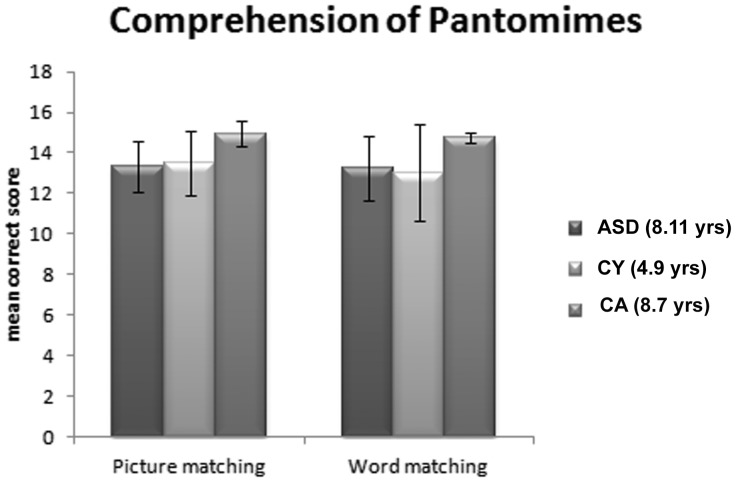
Comprehension of Pantomimes. Mean correct responses and standard deviations in the two tasks requiring a match between the observed pantomime and either a set of pictures or the spoken word of a tool.

In the picture-pantomime matching task, the child is required to select a picture corresponding to the observed pantomime; in the word-pantomime matching task, the child is required to judge whether or not the heard word corresponds to the observed pantomime. As evident from Fig. 3, in both the picture and the word pantomime comprehension tasks, ASD children fare no better than the younger TD sample and worse than the CA matched control children. A mixed-model design ANOVA indicates that the Group Factor (ASD and the two TD samples) is highly significant: (F [2,75] 12,771, p<.0001) as well as the Task Factor (matching of pantomimes with visual and verbal cues) (F [1,75] 7,794, p<.007); there is no interaction of Group by Task (F [1,75] 1,589 p<.211).

## Discussion

Overall, our findings highlight two key aspects: 1) in ASD children the inefficiency of motor cognition is not restricted to imitation, but it cuts across the domains of both production and comprehension of pantomimes; 2) the accuracy of performance in each motor domain can be either worsened or improved, owing to the availability for ASD children of pre-existing motor plans, cued by a facilitating pragmatic context. Clearly, normal intelligence and chronological age advantage do not suffice for ASD children to achieve levels of performance equating even younger TD controls.

Let us first consider the imitation of conventional actions: when the ASD children are allowed the use of objects, they obtain a mean score of 42 (SD = 2,61), but their performances collapse to a low mean score of 30,2 (SD = 5,89) when the imitation of the same actions has to be performed without the use of the actual object. These performances are well below the level of even the younger (4,9 years) control sample, who obtain a mean score of 43,35 (SD = 1,02) and 41,47 (SD = 2,50) in the former and the latter task, respectively. Uneven performances of this magnitude suggest that ASD children find it particularly difficult to select a proper motor plan, when it requires a context-independent feed-forward mode of motor control. It is as if the required action had to be planned anew and the access to the stored motor templates was denied. In this condition, with the context playing no supportive role, ASD children are left with a motor task that is clearly far beyond their possibilities.

The relevance of a favourable external cue can be detected even in the comprehension of actions. In a previous study [36] we showed that ASD children succeed in telling the “what” of an action (e.g.: touching *vs.* grasping), but fail in understanding the “why” of the same action (e.g.: grasping a cup for drinking, or grasping a cup for placing) unless the action is embedded in an environmental context providing external cues to decipher the intention of the observed action. Our two present tasks, requiring the comprehension of pantomimes, reveal that ASD children fail even to grasp the “what” of a pantomime, when the contextual cues are less favourable (as when required to judge the correspondence between an observed pantomime and the heard name of a tool): the statistical analyses confirm that ASD fare significantly worse than their control samples.

A further support to the widespread inefficiency of motor cognition in ASD children is provided by two additional imitation tasks: when our ASD children are required to imitate either oro-facial gestures or non-symbolic finger positions, their performance falls significantly below the accuracy level of TD samples.

It should be noticed, though, that the interpretation of these findings is far from being unproblematic, as a number of experiments failed to document an impairment of imitation in ASD [8], [9], [10] and have explicitly questioned a motor, “domain specific” account, in favour of a more general (e.g. attentive), non imitative account [8], [10]. As to the first point, we notice that all of the ASD patients in the above studies were adult subjects (30 to 40 years of age) with normal IQ scores and that the selected imitation tasks were of an extremely simple nature (opening and closing the mouth [8], or opening and closing a hand [9]. The imitation tasks that we presented were of a more complex nature and required precise sequencing of the motor acts, furthermore the ASD patients in our study were children of 8,7 years of age. We maintain that motor skills in ASD children may change over time both as a consequence of daily life experience as well as of their remedial training. We suspect that it can hardly be denied that one of the key clinical aspects of ASD children is a disorder in motor cognition; the more so, since similar findings continue to emerge from very recent research, reiterating that “impairments in performance and recognition of skilled movements appear to be specific to Autism [37].

In one of the papers holding a critical position toward a “motor account”, the Authors assert that imitation disorders in ASD “may be driven by a lack of attention, or motor sequencing impairments” [8]. As to the first variable, in our study we can hardly call into question general attentional factors, owing to our rigid inclusion criteria for the selected sample of high functioning ASD children, whose mean Full-Scale IQ is 91 (SD 12,02). In addition, by comparing their Full-scale IQ with the control groups’ IQ scores (from the Raven Matrices), we detect significant difference neither between ASD and CY group (t = 0.424, p<.672) nor between ASD and CA group (t = 0,3003, p<.765).

Furthermore, as evident from other studies, when attention is carefully controlled in imitation tasks with very young ASD children, it appears that “there [is] no evidence that either motor or attentional aspects of the tasks contributed to the poorer imitative performance of the children with autism” [11]. As to the second variable, namely the “motor sequencing impairments”, it is precisely one of the components that we maintain can be hampered in ASD children, as part of a broader impairment in the domain of motor cognition. As a matter of fact, the difficulties in the planning and execution of actions surface in their full evidence when ASD children are required to generate a tool-use pantomime, either by a picture or by the name of the tool. Under these constraints, the required action seems to lie far beyond the reach of ASD children and their accuracy in both tasks is significantly below the accuracy level of even the younger TD sample.

An overview of the findings from our study allows some speculations on the role of motor cognition in ASD children. In particular, the concomitant impairment of both production and “comprehension” of actions is suggestive of an early damage to a core component implementing both sides of action representation. We suggest that one such component is represented by the MNM that provides the neural substrate for matching action perception and action execution.

The theoretical relevance of the role played by the MNM in ASD can be fully appreciated when we consider a series of recent experiments that shed new light on the mechanisms that control both execution and pre-cognitive comprehension of motor acts in ASD children. An EMG experiment [38], for instance, has documented that, unlike TD children, high-functioning ASD children are unable to organize their own motor acts in intentional motor chains. In particular, the latter group fail to activate a specific action chain from its very outset, thus being deprived of an internal copy of the whole action *before* the execution thereof. Participants to this study were required both to execute and observe two different actions: a) grasping with the right hand a food item placed on a plate, bringing it into the mouth and eating it; b) grasping a piece of paper placed on the same plate and putting it into a box. During the execution *and* observation conditions of both actions the activity of the mouth-opening mylohyoid muscle (MH) of the participants was recorded using EMG surface electrodes. The results showed that during the execution and observation of the eating action, a sharp increase of MH activity was recorded in TD children, starting well before the food was grasped. No increase of MH activity was present during the observation of the placing action. This means that one of the muscles responsible for the action final goal (opening the mouth to eat a piece of food) is already activated during the initial phases of the action. The motor system anticipates the final goal of the action (to eat), thus directly mapping the action intention, both when the action is executed, or it is observed when performed by others. In contrast with TD children, ASD children showed a much later activation of the MH muscle during eating action execution and *no activation at all* during eating action observation.

The results from Cattaneo et al. [38] reveal that ASD children are impaired in the smooth chaining of sequential motor acts within a reaching-to-grasp-to-eat intentional action. This impairment is mirrored in the action observation condition, and most likely accounts for their difficulty in directly understanding the intention of the observed action when executed by others.

Further aspects of the motor domains, such as action simulation, mimicry and imitation, have been recently explored by a number of studies, all confirming a deep impairment of the core mechanisms of motor cognition in ASD children. Théoret and colleagues [39], by means of Transcranial Magnetic Stimulation (TMS) demonstrated that, unlike healthy controls, ASD individuals did not show TMS-induced hand muscle facilitation during hand action observation. Electroencephalographic (EEG) studies showed that ASD individuals, in contrast with healthy controls, did not show mu frequency attenuation over the sensory-motor cortex during action observation, a sign of MNM activation [40], [41]. Bernier et al. [42] demonstrated imitative deficits in ASD individuals, and a positive correlation between the severity of such deficits and the reduced attenuation of mu-rhythm over the motor cortex during action observation, thus suggesting a relationship between imitation deficits and a malfunctioning MNM in ASD individuals.

However, it has been shown that mu attenuation is sensitive to the degree of familiarity between agent and observer: when a sibling or someone related with the ASD individuals performed the observed action, mu attenuation could be detected [43]. In fact, as pointed out by Ramachandran and Pineda [43], ASD children display improved communication skills, increased rate of physical contact and eye contact, as well as improved social interaction skills when they interact with familiar as opposed to unfamiliar individuals. Such findings point to an emotional gating of the MNM still to be thoroughly explored both in TD as well as in ASD individuals. An fMRI study [44] explores the capacity to imitate facial expressions of basic emotions in ASD children; the results from this study indicate that ASD children do not activate properly the pars opercularis of the inferior frontal gyrus either during observation or during imitation. Furthermore, experiments with EMG recording [45], [46] show that ASD children, unlike healthy controls, do not produce automatic mimicry of the facial expression of basic emotions, although they can voluntarily do it upon request.

The simultaneous impairment of action imitation, production and comprehension of pantomimes, as revealed by the present data, suggest that the process of constructing an action motor representation is deeply impaired in ASD children. Interestingly, the neuro-functional correlates of tool use pantomimes are associated with the activation of the left intra-parietal cortex and dorsolateral frontal cortex [47], namely with those brain structure involved in the MNM circuitry. Furthermore, a recent fMRI study pinpoints a link between the dysfunction of emotion recognition and motor system by showing that ASD subjects “failed to activate amygdala, inferior frontal gyrus and premotor cortex when viewing fearful actions,” [48].

Overall, the wide range of deficits shown by ASD children in different motor domains, converge toward an impairment of a basic neurophysiologic mechanism that allows children (and adults alike) to “seeing others as a template of the self “[4]. We suggest that the broad range of motor disorders observed in ASD children can find a theoretically principled account in the impairment of the MNM. Indeed, we suggest that the MNM constitutes the neural substrate for matching both a pre-cognitive “understanding” of actions and the planning (and execution) of intentional actions. Accordingly, we suspect that an early (and profound) damage to this system is likely to be reflected in a deficient organization of motor representation, besides disrupting the development of the links between action, intentional attunement [49] and the growth of a simulating social mind [27], [50], [51].
